# Optimizing PCR primers targeting the bacterial 16S ribosomal RNA gene

**DOI:** 10.1186/s12859-018-2360-6

**Published:** 2018-09-29

**Authors:** Francesco Sambo, Francesca Finotello, Enrico Lavezzo, Giacomo Baruzzo, Giulia Masi, Elektra Peta, Marco Falda, Stefano Toppo, Luisa Barzon, Barbara Di Camillo

**Affiliations:** 1 Department of Information Engineering, University of Padova, Padova, Italy; 2 Biocenter, Division of Bioinformatics, Medical University of Innsbruck, Innsbruck, Austria; 3 Department of Molecular Medicine, University of Padova, Padova, Italy

**Keywords:** 16S rRNA sequencing, Primer design, Multi objective optimization

## Abstract

**Background:**

Targeted amplicon sequencing of the 16S ribosomal RNA gene is one of the key tools for studying microbial diversity. The accuracy of this approach strongly depends on the choice of primer pairs and, in particular, on the balance between efficiency, specificity and sensitivity in the amplification of the different bacterial 16S sequences contained in a sample. There is thus the need for computational methods to design optimal bacterial 16S primers able to take into account the knowledge provided by the new sequencing technologies.

**Results:**

We propose here a computational method for optimizing the choice of primer sets, based on multi-objective optimization, which simultaneously: 1) maximizes efficiency and specificity of target amplification; 2) maximizes the number of different bacterial 16S sequences matched by at least one primer; 3) minimizes the differences in the number of primers matching each bacterial 16S sequence. Our algorithm can be applied to any desired amplicon length without affecting computational performance. The source code of the developed algorithm is released as the mopo16S software tool (Multi-Objective Primer Optimization for 16S experiments) under the GNU General Public License and is available at http://sysbiobig.dei.unipd.it/?q=Software#mopo16S.

**Conclusions:**

Results show that our strategy is able to find better primer pairs than the ones available in the literature according to all three optimization criteria. We also experimentally validated three of the primer pairs identified by our method on multiple bacterial species, belonging to different genera and phyla. Results confirm the predicted efficiency and the ability to maximize the number of different bacterial 16S sequences matched by primers.

**Electronic supplementary material:**

The online version of this article (10.1186/s12859-018-2360-6) contains supplementary material, which is available to authorized users.

## Background

Targeted amplicon sequencing of the ribosomal small subunit, 16S ribosomal RNA gene (16S rRNA) is a common approach to investigate the diversity of microbial communities in a site [1, 2]. The 16S rRNA gene is present in all prokaryotes and contains both fast-evolving regions, which can be used to classify organisms at different taxonomic levels, and slowly-evolving regions, which are relatively conserved throughout different species. The slowly-evolving regions can be used to design broad-spectrum primer pairs for polymerase chain reaction (PCR) amplification, which in turn can be used to isolate species-specific fast-evolving regions. A primer pair is composed of a *forward* and a *reverse* primer: the former is meant to match the sense sequence of the bacterial 16S, while the latter should match the antisense sequence [1]. The accuracy of 16S rRNA sequencing strongly depends on the choice of the primer pairs. Many of the current bacterial 16S primers have been designed from sequence data obtained from in vitro cultured species, even though environmental microbiologists estimate that less than 2% of bacteria can be cultured in the laboratory. However, our knowledge over unculturable bacterial sequences is rapidly growing thanks to Next-Generation Sequencing (NGS), a technology that is continuously evolving and improving [3]. As a consequence, several 16S sequence databases have been created and are being maintained up to date by the scientific community [4–6]. There is thus the need for automated methods that leverage such newly available information in the design and update of bacterial 16S primers.

Since the 16S gene sequence is similar but not identical in different organisms, degenerate primers are used for 16S rRNA sequencing. A primer set is called *degenerate* when it is used as a mixture of oligonucleotide molecules that contain different nucleotides in defined positions. A pair of degenerate primers can be naturally expanded into a set of non-degenerate primer pairs, whose elements are obtained by assigning all possible combinations of values to the degenerate nucleotides of the original pair. We define such a set of non-degenerate primer pairs a *primer-set-pair* (Table 1).

**Table 1:** Example of the mapping from a pair of degenerate primers to a primer-set-pair

DG (forward)	DG (reverse)	NDG (forward)	NDG (reverse)
ACGT**H**ACGT	**R**ACGT**Y**ACGT	ACGTAACGT	AACGTCACGT
		ACGTCACGT	AACGTTACGT
		ACGTTACGT	GACGTCACGT
			GACGTTACGT

*DG* degenerate primer, *NDG* non-degenerate primer

The degenerate bases are shown in bold

An optimal primer-set-pair should exhibit several properties:

- Maximize experimental efficiency and specificity, in terms of how much a primer pair is able to amplify the selected DNA sequence, and not others, during PCR amplification. Efficiency and specificity depend on a number of parameters intrinsic to the PCR method, which need to be set in order to guarantee the success of the reaction. Key parameters are the primer length, the amplicon length, the number and position of mismatches with respect to the template, the primer GC-content, and the ability of primers to produce secondary structures by inter- or intra-molecular interactions [7]. In the following, for the sake of conciseness, we refer to this objective with the term *efficiency*.
- Maximize *coverage*, in terms of the fraction of all bacterial 16S sequences from different species that are targeted by at least one forward and one reverse primer from the primer-set-pair.
- Minimize primer *matching-bias*, in terms of differences in the number of combinations of primers from the forward and reverse sets matching each bacterial 16S.

In the literature, the majority of the approaches for automated primer design for a set of reference sequences are based on multiple alignment of the set of sequences. Among these, Linhart and Shamir [8] formulate the problem as the Degenerate Primer Design problem and propose a dynamic programming solution, implemented in the HYDEN software. An improvement of the HYDEN software is proposed by Hugerth et al. [9] as the DegePrime software. None of these approaches account for primer efficiency, which instead is taken into account by Brodin et al. [10] in the PrimerDesign software, as a set of constraints on admissible primer pairs. Multiple alignment, however, is based on heuristic approaches [11] and is inherently ineffective in producing a correct final alignment when thousands of sequences are involved in the process, especially when sequences show a certain degree of heterogeneity as in the case of 16S.

Multiple alignment of the 16S bacteria sequences from the Ribosomal Database Project (RDP) [5] is used by Wang and Qian [12] to identify conserved fragments useful for primer design, but the approach focuses just on single primers and does not extend the analysis to primer pairs. Finally, the SPYDER software for 16S primer design and assessment [13] exploits the RDP Probe Match tool to quickly assess coverage of candidate primer pairs, but the primer design has to be manually carried out by the user, rather than automated by the software.

In this work, we propose an algorithm for optimizing the primer choice, which searches within the set of all possible primer-set-pairs for those simultaneously exhibiting high efficiency and coverage and low matching-bias. The novelty of our approach is many-fold. First, by formulating coverage, efficiency and matching-bias as optimization criteria, we allow the user to explicitly model the trade-off between the three competing objectives. Second, we consider for the first time minimal matching-bias among the characteristics that a good primer-set-pair must exhibit. While efficiency and coverage are usually taken into account when designing a primer set, matching-bias is seldom considered in the literature. However, it should be taken into account in quantitative studies, where the objective is to quantify the relative abundance of the different species, and the presence of species matched by more combinations of forward and reverse primers may lead to unwanted amplification biases. Third, by relying on primer-to-sequence alignment, rather than on multiple alignment, we avoid potential artefacts in the results due to incorrect final alignment when thousands of sequences are involved in the process. Fourth, we remove the constraint that the sets of forward and reverse primers should be summarizable as a pair of degenerate primers: indeed, the inclusion of degenerate base sites in primer design may lead to inefficient target amplification, due the presence of mismatches between primers and target sequences [14]. In addition, the use of degenerate primers might lead to low-reproducibility in primer synthesis and thus biases among different primer batches. By avoiding degenerate primers, we thus provide the user with more control over what is actually amplified and over possible biases.

Our approach exploits the bacterial sequence knowledge available in public databases such as GreenGenes [4], the probeBase 16S primers database [15], recently updated after a comprehensive literature survey [16], and SILVA [6]. As an example of application, we present the optimization of primer choice for amplicons in the range 700–800 bp, but the procedure is general and can be applied to any desired amplicon length and representative bacteria population.

In silico results show that our strategy is able to find better primer-set-pairs than the ones available in the literature according to all three optimization criteria. Furthermore, experimental validation demonstrates that the optimal primer-set-pairs are suitable for the amplification of 16S rRNA from a variety of bacterial species belonging to different genera, thus confirming the predicted efficiency, wide coverage and low matching-bias.

## Methods

### Problem constraints

As stated in the previous paragraph, an optimal primer-set-pair should simultaneously maximize efficiency and coverage and minimize matching-bias. In the following, we describe how we quantitatively encoded these constraints.

### Efficiency

The perfect primer-set-pairs should satisfy several constraints, aimed at improving PCR efficiency and specificity [7]. However, concurrently satisfying all constraints is often impractical and most state-of-the-art primers violate one or more constraints [16]. We thus decided to introduce efficiency as an optimization score, encoding many of the constraints as fuzzy score functions. More precisely, we defined our efficiency score as the sum of ten score terms: seven fuzzy score terms related to single-primer efficiency constraints, averaged across all primers in the primer-set-pairs, plus three score terms related to the efficiency of the primer-set-pairs as a whole. Since all terms are meant to vary between 0 and 1, the optimization score ranges from 0 (minimal efficiency) to 10 (maximal efficiency).

Broadly speaking, our fuzzy score counts 1 for each constraint that is perfectly satisfied, or, alternatively, a value between 0 and 1 depending on how close the primer is to the constraint limit. As an example, consider the primer melting temperature, *T*_*m*_. *T*_*m*_ should be greater than or equal to 52 degrees in a perfect primer [7], but 51 is still tolerable, albeit not ideal. In this case, our fuzzy scoring function assigns 1 to temperatures of 52 degrees or greater, 0 to temperatures of 50 degrees or less and considers a linear increasing function between 50 and 52 degrees. Each term is precisely described in what follows.

The 7 single-primer score terms are:

1. *Melting temperature*: the melting temperature *T*_*m*_ of a primer is computed with the nearest-neighbour formula [17]. The score term is 1 if *T*_*m*_ ≥ 52, 0 if *T*_*m*_ ≤ 50 and (*T*_*m*_ *-* 50)*/*2 if 50 *< Tm <* 52.
2. *GC-content*: GC-content is the fraction *f*_*GC*_ of base pairs in the primer sequence equal to either G (guanine) or C (cytosine). The score term is 1 if 0*.*5 ≤ *f*_*GC*_ ≤ 0*.*7, 0 if *f*_*GC*_ *>* 0*.*7 or *f*_*GC*_ *<* 0*.*4 and (0*.*5 *- f*_*GC*_)*/*0*.*1 if 0*.*4 ≤ *f*_*GC*_ *<* 0*.*5.
3. *3′-end stability - score term 1*: two score terms are defined concerning 3*′*-end stability. The first term is 0 if the last three bases of the primer consist entirely of As (adenines) and Ts, (thymines) and 1 otherwise.
4. *3′-end stability - score term 2*: the second score term is 0 if the last 5 bases contain more than 3 Cs or Gs, and 1 otherwise.
5. *Homopolymers*: a homopolymer is a sequence of identical nucleotides. The score term is 1 if there are no homopolymers longer than 4 nt, 0.5 if there are no homopolymers longer than 5 nt, and 0 if there is at least a homopolymer longer than 5 nt in the sequence.
6. *Self-dimers*: the presence of self-complementary regions between couples of identical primers can lead to the generation of self-dimers. Considering the maximum number of matches in a gap-free alignment between a primer with its reverse complement, *max*_*M*_, the score term is 1 if *max*_*M*_ ≤ 8, 0 if *max*_*M*_ *≥* 11 and (11 *- max*_*M*_)*/*3 if 8 *< max*_*M*_ *<* 11.
7. *Hairpins*: a hairpin can be formed in the presence of self-complementarity within the primer sequence, especially at its 3*′*-end. The score term is 0 if, for at least one gap-free alignment between the primer and the reverse complement of its 3*′*-end, both the last nucleotide and 3 or more of the 4 preceding nucleotides match, and 1 otherwise.

The 3 primer-set-pairs score terms are defined as follows:

1. *Melting temperature range*: the melting temperature range Δ*T*_*m*_ of a primer-set-pair is computed as the maximum minus the minimum of the melting temperatures of all primers in the set pair. The score term is 1 if Δ*T*_*m*_ ≤ 3, 0 if Δ*T*_*m*_ *≥* 5 and (5 *-* Δ*T*_*m*_)*/*2 if 3 *<* Δ*T*_*m*_ *<* 5.
2. *Dimers*: we consider the maximum number of matches *max*_*M*_ across all possible alignments between all possible combinations of forward and reverse primers from a primer-set-pair. The score term is 1 if *max*_*M*_ ≤ 8, 0 if *max*_*M*_ *≥* 11 and (11 *- max*_*M*_)*/*3 if 8 *< max*_*M*_ *<* 11.
3. *Amplicon length range*: due to the known reduction of PCR efficiency with increasing amplicon length [7], we want the lengths of the generated amplicons to lie in a narrow range. We especially want to avoid amplicons much shorter than the target length, since they would be over-amplified with respect to the others. However, we want to be able to tolerate a small fraction of outliers, in order to avoid penalizing potentially valuable primer-set-pairs due just to a few rare sequences. Given a representative set of bacterial 16S sequences, called “reference set” from now on, we consider the difference Δ_*amplen*_ between the median and the first percentile of amplicon lengths across all possible amplicons, formed by matching all combinations of forward and reverse primers from the set pair with the reference set. The score term is 1 if Δ_*amplen*_ ≤ 50 nucleotides, 0 if Δ_*amplen*_ *≥* 100 and (100 *-* Δ_*amplen*_)*/*50 if 50 *<* Δ_*amplen*_ *<* 100.

The choice of the scoring criteria and the default threshold are based on previous literature [7, 18, 19]. However, both the thresholds and the fuzzy tolerance intervals can be set by the user differently from the default and according to his/her experimental needs by specifying the desired values as input parameters when calling the command line tool.

### Coverage

The coverage score is defined as the number of 16S sequences matched by at least one primer. Given the sequences of a primer and of a bacterial 16S, we define *seed* the last 5 nucleotides at the 3*′*-end of a primer and we consider a 16S sequence as matched by the primer if a region of the 16S sequence exists that matches i) the *seed* of the primer exactly; and ii) the remainder of the primer with at most 2 mismatches [20]. A 16S sequence from a reference set is considered *covered* by a primer-set-pair if at least one forward and one reverse primer in the primer-set-pair match the sequence. Since PCR efficiency decreases with amplicon length, we impose a further constraint: given a primer-set-pair and a reference set of 16S sequences, we estimate the *target amplicon length* as the median of the lengths of all amplicons obtained by matching all combinations of forward and reverse primers from the primer-set-pair with the reference set. We then consider as not covered all 16S reference sequences whose amplicon length differs more than 100 nucleotides (either longer or shorter) from the target length.

### Matching-bias

Given a reference set of 16S sequences and a primer-set-pair, the third optimization score measures the variability of the number of combinations of forward and reverse primers matching each 16S reference sequence. Coverage variability due to matching bias should be minimized, or at least accounted for, when the study is meant to quantify the relative abundances of the different bacterial species, because of the amplification bias towards the species covered by more combinations of forward and reverse primers. As a measure of matching-bias, we exploit the coefficient of variation of the coverage across the target sequences, computed as the standard deviation over the mean of the number of combinations matching each sequence.

### Reference set of 16S sequences, preparation and annotation

To optimize the three scores above, we rely on a representative set of bacterial 16S sequences extracted from a public 16S sequence database, GreenGenes [4]. The GreenGenes 16S sequence database is organized in Operational Taxonomic Units (OTUs), which are nested clusters of sequences in the database, organized at different levels of inter-cluster similarity. For each level of similarity, a reference sequence is associated to each cluster, maximally similar to all other sequences in the same cluster [4]. The set of reference sequences can thus be considered a representative subset of the entire sequence database, becoming more and more accurate for increasing levels of inter-cluster similarity (and, thus, number of reference sequences). We chose an 85% inter-cluster similarity level as a good trade-off between representativeness and complexity, corresponding to a set of 5088 representative sequences to be used to assess the optimization criteria.

Albeit very sensitive in annotating the Bacteria and Archaea domains, the GreenGenes taxonomy is not designed to distinguish sequences belonging to eukaryotes or viruses. For this reason, we decided to re-annotate 16S bacterial sequences leveraging the original NCBI taxonomy [21] to accurately identify, among the representative sequences, only the ones belonging to the Bacteria domain. Since domain information is missing from the NCBI annotation for around 20% of the sequences, we designed an ad hoc procedure to identify bacterial sequences among these. The procedure is described in detail in the Supplementary Materials (see Additional file 1). We conservatively chose to consider only the sequences annotated as bacteria both in our curated, NCBI-based annotation and in the original GreenGenes annotation. This resulted in a set of 4573 representative 16S sequences belonging to the Bacteria domain.

### Optimization algorithm

Since the problem of optimal primers choice requires the simultaneous optimization of different competing scores, it can be cast as a multi-objective optimization problem, where the search space is the set of all possible primer-set-pairs and a scoring function, or optimization criterion, can be defined so to maximize efficiency and coverage and minimize matching-bias. When more than one criterion needs to be optimized concurrently, but the objectives to be optimized are conflicting, one is usually not interested in a single solution, but rather in the set of *Pareto* optimal solutions, i.e. in the set of solutions for which none of the objectives can be improved without sacrificing at least one other objective [22]. The result of multi-objective optimization is no longer a unique optimal primer-set-pair, as in single-objective optimization, but rather a collection of primer-set-pairs that are not worse than any other primer-set-pair and strictly better according to at least one of the criteria. More precisely, for the tri-objective optimization problem of maximizing the efficiency (E) and coverage (C) optimization scores and minimizing the matching-bias (M) score, as defined in the previous section, candidate primer-set-pairs are evaluated according to an objective function vector **f** = (*f* _*E*_ *; f* _*C*_ *; f*_*M*_). Given two primer-set-pairs *p* and *p′*, we say that *p dominates p′* (*p ≺ p′*) if and only if **f** (*p*) *≠* **f** (*p′*), *f*_*E*_ (*p*) *≥ f*_*E*_ (*p′*), *f*_*C*_ (*p*) *≥ f*_*C*_ (*p′*) and *f*_*M*_ (*p*) *≤ f*_*M*_ (*p′*). If no *p′* exists such that *p′ ≺ p*, the primer-set-pair *p* is called *Pareto-optimal*. In this context, the goal of optimal primers choice is to determine (or approximate) the set of all Pareto-optimal primer-set-pairs, whose image in the tri-objective space is called the *Pareto front* [22].

To search for the optimal Pareto front we rely on the two-phase iterated best improvement local search approach proposed by Dubois-Lacoste et al. [23] and effectively exploited in Sambo et al. [24] and Borrotti et al. [25] for the optimal multi-objective design of experiments.

Local search starts from an initial solution and iteratively refines it by applying small local changes and assessing each time their effect on solution quality; it stops when no further local changes can improve the solution. The process is iterated from several different starting points and the best solution ever found is returned, as an approximation of the unknown optimum [26]. A common extension of local search to the multi-objective case is to start from a set of initial Pareto solutions, sample one solution from the front, optimize with local search a random *scalarization* of the problem, i.e. a linear combination of the optimization scores with weights sampled uniformly at random from the unit simplex, update the Pareto front and iterate until a termination condition is met [23].

The procedure MULTI-OBJECTIVE-SEARCH, whose pseudo-code is reported in what follows, receives as inputs the desired range of amplicon lengths (*range*_*amplen*_), a representative set of 16S sequences (*repset*), an initial set of (possibly degenerate) primer pairs (*init*) and the number of restarts (*n*_*res*_). The procedure begins by selecting from *init* all possible primer pairs with the desired amplicon length, primer length (between 17 and 21 nucleotides) and target domain (*Bacteria* or *Universal*).

Degenerate primer pairs are converted to non-degenerate primer-set-pairs and added to an archive. The procedure then iterates *n*_*rest*_ times, each time sampling a random primer-set-pair *p*_*start*_ from the Pareto front and a random vector ***α*** of relative weights for the optimization scores, with weights sampled uniformly from the unit simplex; the procedure, then, solves a *scalarization* of the multi-objective problem, i.e. a single-objective problem in which a linear combination of the three objectives with relative weights ***α*** is maximized, and adds the result to the archive. To this purpose, efficiency, coverage and matching-bias scores are normalized to their maximum, so that each normalized score ranges between 0 and 1, and matching-bias is redefined as 1 – matching-bias, so that it can be maximized as the other scores.

MULTI-OBJECTIVE-SEARCH (*range*_*amplen*_ *; repset ; init ; n*_*rest*_)

1 Select all (possibly) degenerate primer pairs from the set *init* with amplicon length in *range*_*amplen*_

2 Add to *archive* the corresponding non-degenerate primer-set-pairs

3 **for** *r* = 1 **to** *n*_*rest*_

4  *pf* = PARETO-FRONT(*archive*)

5  Sample *p*_*start*_ from *pf*

6  Sample ***α*** from [0*,* 1]^3^, with Σ_*i*_ *α*_*i*_ = 1

7  *p* = LOCAL-SEARCH(*p*_*start*_ *,* ***α*** *, repset*)

8  Add *p* to *archive*

9 **return** *archive*

Single-objective optimization is obtained using the Best Improvement Local Search algorithm [26]: starting from an initial primer-set-pair, the LOCAL-SEARCH algorithm cycles through the primers of the set-pair and, for each primer, scans its *neighbourhood*, i.e. the set of all possible *local perturbations* of the primer. Local perturbations consist in all possible flips of one nucleotide (assessing the three other possible bases) and all possible additions and removals of one nucleotide at the extremities. The search in the solution space is performed with the *best improvement local search* approach: after generating the entire neighbourhood as explained above, the algorithm selects the best neighbour perturbation, starts from it to generate the next neighbourhood, and iterates until it reaches a solution for which no better neighbour perturbation can be found. The procedure terminates when no further local improvements can be applied to any primer in the primer-set-pair. The WEIGHTED-SCORE function computes the three optimization scores from a primer-set-pair and the representative set, multiplies the scores by the relative weights ***α*** and returns the sum of the results.

We developed a software tool implementing our approach and released it under the GNU General Public Licence as the mopo16S software tool (Multi-Objective Primer Optimization for 16S experiments) at http://sysbiobig.dei.unipd.it/?q=Software#mopo16S. mopo16S is implemented as a multithreading C++ command line tool; the software tool relies on the efficient algorithms and data structures from the SeqAn library [27] and uses the openMP library [28] for multithreading.

LOCAL-SEARCH(*p*_*start*_ *,* ***α*** *, repset*)

1 *p*_*best*_ = *p*_*curr*_ = *p*_*start*_

2 *score*_*best*_ = *score*_*curr*_ = WEIGHTED-SCORE(*p*_*curr*_*,* ***α*** *, repset*)

3 **while** improvement

4  **for** *i* = 1 to *|p*_*curr*_*|*

5  *pr*_*i*_ = *i*-th primer of *p*_*curr*_

6   **for** *p*_*new*_ = *p*_*curr*_ with all possible additions and removals of a base at the extremities and replacements of a base of *pr*_*i*_

7    *score*_*new*_ = WEIGHTED-SCORE(*p*_*new*_ *,* ***α*** *, repset*)

8    **if** *score*_*new*_ *> score*_*best*_

9    *p*_*best*_ = *p*_*new*_

10    *score*_*best*_ = *score*_*new*_

11   *p*_*curr*_ = *p*_*best*_

12 **return** *p*_*curr*_

### State-of-the-art primer pairs as initial solutions

We selected the online database probeBase [15, 16] as a source of candidate primer-set-pairs to be used as initial solutions by mopo16S. The database contains more than 500 pairs of (possibly degenerate) primers and reports for each primer its sequence, the strand and position at which it matches the reference 16S *Escherichia coli* gene, and the target domain for which it is designed (being either Bacteria, Archaea or Universal).

Given a desired range for the target amplicon length as input of mopo16S, we selected all primer pairs from the probeBase database satisfying all the following properties:

- Amplicon length in the desired range;
- Length of both primers greater than or equal to 17 nt and smaller than or equal to 21 nt;
- Bacteria or Universal target domain of both primers.

Since our approach is to work with sets of non-degenerate primers, in case of degeneracies in either the forward or the reverse primer, we substitute the degenerate primer with a corresponding set of non-degenerate primers, obtained by assigning all possible combinations of values to the degenerate nucleotides in the primer. An example of this procedure is given in Table 1.

We computed the three scores for each of the primer-set-pairs and identified, among these, the primer-set-pairs forming the initial Pareto front.

## Results

**Fig. 1:**
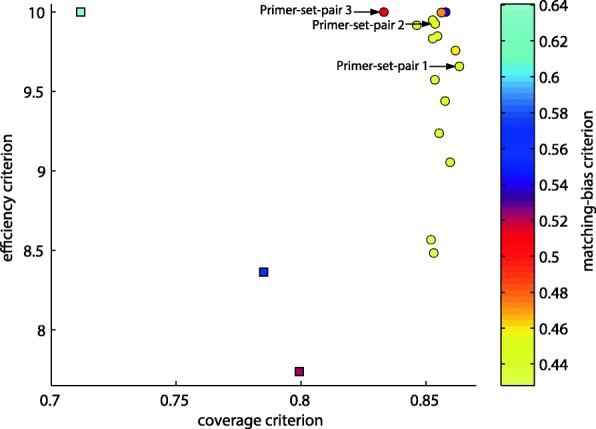
Representation of the efficiency, coverage and matching-bias optimization criteria for the Pareto front. Efficiency is represented on the y-axis, coverage on the x-axis and matching-bias using color shading. The initial primer-set-pairs are represented as squares; the primer-set-pairs after multi-objective optimization are represented as circles

We present a case study of optimal primer choice procedure targeting amplicons in the range of 700–800 bp. From the set of initial primer-set-pairs in the probeBase database, we identified 37 set pairs satisfying all the required properties and having reference amplicons in the desired range. Exploiting the 4573 16S sequences of the GreenGenes bacterial OTUs as representative set (see the Methods section), we computed the three scores for each of the primer-set-pairs and identified three primer-set-pairs forming the initial Pareto front, represented as squares in Fig. 1.

We then executed mopo16S, launching 20 runs of the MULTI-OBJECTIVE-SEARCH algorithm, each with 20 restarts, for a total of more than 33,000,000 function evaluations. The lists of solutions returned by the 20 runs are quite heterogeneous, having a mean Jaccard index (size of intersection over size of union) between each pair of lists equal to 0.007. The software collected the results of all the 20 runs in a single archive and computed the new Pareto front, represented as circles in Fig. 1 (note that the ideal points should be bright yellow and located to the top right corner of the figure). mopo16S completed its execution in less than 9 min, using less than 900 MB of RAM and up to 4 threads on a desktop workstation equipped with a 3.3 GHz Intel® Core™ i5–2500.

The initial primer-set-pairs, chosen as the best-performing primer-set-pairs extracted from the probeBase database (indicated as squares in Fig. 1), are outperformed by all primer-set-pairs obtained by our approach (circles in Fig. 1) according to at least two criteria and, some of them, according to all three criteria. In particular, one of the initial primer-set pairs considered in the probeBase database (cyan square in Fig. 1) has maximum efficiency (score 10), but the lowest coverage and the highest matching-bias compared to all the other solutions. The other two initial primer-set pairs, instead, are outperformed by all the new solutions according to all three criteria, with a single exception of a solution with equal matching-bias (purple square and purple circle in Fig. 1).

### *In-silico* validation

From the optimal primer-set-pair solutions (circles) in Fig. 1, we selected the three set pairs marked with arrows for further inspection. The forward primers of all three pairs align to the reference 16S sequence of the *Escherichia coli* bacterium between hypervariable regions V2 and V3, at positions 355–358, and all three reverse primers align between regions V6 and V7, at positions 1059–1063, thus resulting in amplicon lengths between 701 and 708 nucleotides. The complete sequence of each forward and reverse primer is reported in Table 2. Each primer-set-pair was compared to the human genome to exclude nonspecific amplification of human sequences. Primer sequences were compared to the GRCh38 human genome with ssearch36 [29], allowing no gaps and up to 2 mismatches, consistently with the *Coverage* constraints. None of the possible primer pairs amplifies a region of the human genome shorter than 4000 nt, which is 5.6-fold the length of the amplicons generated in the bacterial 16S rRNA.

**Table 2:** Complete sequence of each forward and reverse primer of the three selected primer-set-pairs

	Forward	Reverse
Primer-set-pair 1	TCCTACGGGAGGCAGCA, TCCTACCGGAGGCACCA, TCCTACGGGCGAATGCAG, CCTACGCGAGGCTGCAA, CCTACGCGAGGCAGCAA, CCTACGGAAGGCAGCAG, CCTACGGGTGGCTGCAG, CTACGGTGGGCTGCAGT	TCACGGCACGAGCTGAC, GACACGAGCTGACGACA
Primer-set-pair 2	TCCTACGGGAGGCAGCA, TCCTACCGGAGGCACCA, TCCTACGGGTGGTTGCAG, TCCTACGGAAGGCAGCAG, CCTACGCGAGGCAGCAA, CCTACGCGAGGCTGCAA, CCTACGGGCGAATGCAG, CTACGGTTGGCTGCAGT	TCACGGCACGAGCTGAC, ACGACACGAGCTGACGAC
Primer-set-pair 3	CTCCTACGGAAGGCAGCA, TCCTACGGGAGCCTGCA, TCCTACGGAAGGGTGCAG, CCTACGGGTTGCAGCAG, CCTACGCGAGGCAGCAA, CCTACGCGTGGTTGCAG, TCTACGGACGGCAGCAA, CTACGTGCGGTTGCAGT	ACGACACGAGCTGACGA, ACGACACGAGCTGACAAC, CACCACGAGCTGACGAC, CAACACGAGCTGACGAGAG

The efficiency, coverage and matching-bias of the three primer-set-pairs computed on the representative set are reported in the first three rows of Table 3. In order to assess how our new primer-set-pairs perform on much broader and complete datasets, we computed coverage and matching-bias of the three primer-set-pairs on the 195,279 16S sequences of the GreenGenes 99% bacterial OTUs and on the 464,618 bacterial 16S sequences of the SILVA SSU Ref 119 Non Redundant (NR) set, obtained by applying a 99% identity criterion to remove highly similar sequences. Results are shown in Table 3 (efficiency is not reported since it does not depend on the considered dataset) and confirm the performance obtained on the representative set. Slightly improved results might depend on the numerosity of the clusters associated to highly representative reference sequence (see paragraph “Reference set of 16S sequences, preparation and annotation”).

**Table 3:** Numerical values of the efficiency, coverage and matching-bias scores for the three selected primers assessed on GreenGenes and SILVA reference sequences

Representative set	Score	Primer-set-pair 1	Primer-set-pair 2	Primer-set-pair 3
GreenGenes 85%	Efficiency	9.66	9.93	10
	Coverage	0.863	0.854	0.833
	Matching-bias	0.45	0.44	0.51
GreenGenes 99%	Coverage	0.969	0.963	0.954
	Matching-bias	0.22	0.22	0.27
SILVA 99%	Coverage	0.974	0.969	0.962
	Matching-bias	0.20	0.19	0.24

The scores have been computed with respect to the GreenGenes 85% representative set, the GreenGenes 99% set and the SILVA 99% not redundant set. Efficiency ranges from 0 to 10 and coverage ranges from 0 to 1

### Experimental validation

**Fig. 2:**
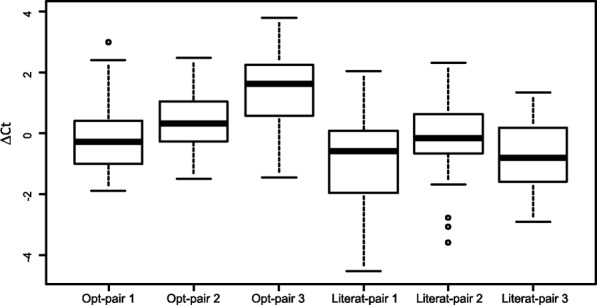
Boxplots of values demonstrating amplification of 16S DNA from bacteria isolates. Primer sets 1, 2 and 3 (Table 2) and three primer pairs from the literature (Forward: Bact-0008-b-S-2 - Reverse: Bact-0785-a-A-21; Forward: Bact-0347-a-S-19; Reverse: Bact-1028-b-A-19; Forward: Bact-0337-a-S-20; Reverse: Bact-1046-a-A-19) were used as real-time PCR primer sets on a panel of bacteria isolated from clinical specimens, including representatives of common Gram-positive and Gram-negative human pathogens belonging to different genera and phyla (Additional file 1: Table S2). ΔCt values were calculated as the difference between the mean of threshold cycle (Ct) values calculated for each sample using different primer-pairs and the Ct value obtained using a specific primer-pair. Positive ΔCt values indicate higher efficiency than average; negative ΔCt values indicate lower efficiency than average

The three primer-set-pairs individuated by mopo16S were also evaluated in a panel of bacteria isolated from clinical specimens, including representatives of different phyla within the Bacteria domain (Additional file 1: Table S1), and compared with three non-optimized primer sets, used as controls, selected among those used to initialize mopo16S and reported by Klindworth et al. [16] (Forward: Bact-0008-b-S-20 - Reverse: Bact-0785-a-A-21; Forward: Bact-0347-a-S-19; Reverse: Bact-1028-b-A-19; Forward: Bact-0337-a-S-20; Reverse: Bact-1046-a-A-19). Bacteria were isolated as pure culture in standard culture media and identified by automated biochemical testing and MALDI-TOF analysis on Vitek 2 and Vitek MS Systems, respectively (BioMerieux, Marcy l’Etoile, France). Nucleic acids were purified from bacteria by using MP 96 DNA SV kits on a MagNA Pure 96 System workstation (Roche, Basel, Switzerland), quantified and diluted in order to achieve approximately the same final concentration. Primer efficiency was evaluated by real-time PCR using SYBR Green I reagent on Real-time PCR on a 7900HT Fast Real-Time PCR System (ThermoFisher Scientific, Carlsbad, CA, USA) with the following steps: 10 min at 95 °C, 35 cycles of denaturation for 30 s at 95 °C, annealing at the selected target temperature for 60 s (60 °C for set 1 and control 3, 56 °C for sets 2 and 3 and control 1 and 2), and extension at 72 °C for 90 s. The specificity of the amplification product was checked by melting curve analysis, which showed no non-specific amplification of human genomic DNA with any of the primer sets under evaluation (Additional file 1: Figure S1). Amplification efficiency and correlation between threshold cycle and target quantity in the sample were demonstrated by amplification of serial dilutions of reference samples. Results of real-time PCR amplification of the panel of bacteria isolates demonstrated that the three PCR primer sets are suitable for the amplification of 16S rRNA from a variety of bacterial genera from different families and phyla, thus confirming the predicted efficiency and wide coverage. Figure 2 shows the boxplots of the ΔCt values calculated as the difference between the mean of threshold cycle (Ct) values calculated across different primer pairs on a specific sample and the Ct value on the same sample obtained with a specific primer-pair. Since Ct levels are inversely proportional to the amount of target nucleic acid in the sample, positive ΔCt values indicate higher efficiency than average; negative ΔCt values indicate lower efficiency than average. Comparison of amplification efficiency based on threshold cycle values showed that optimal primer-set-pairs 2 and 3 outperform literature primers (two-sided paired t-test *p*-value lower than 0.05 for all comparisons with literature primer-sets) with primer-set-pair 3 as the best performer (Fig. 2). Optimal primer-set-pair 1 shows comparable experimental efficiency with literature primers. Cycle sequencing of PCR products obtained with primer-set-pair 3, followed by phylogenetic analysis on the leBIBI-PPF webserver (Jean-pierre Flandrois, Guy Perrière, Simon Penel, Bénédicte Lafay and Manolo Gouy, University of Lyon, 1. http://umr5558-bibiserv.univ-lyon1.fr/lebibi/PPF-in.cgi) was performed to check the ability to identify bacteria at genus and species levels. All the samples under evaluation were classified at genus level with scores > 0.99 according to Shimodaira and Hasegawa test [30, 31], while classification at species level was achieved in > 50% of cases.

## Discussion

In this paper, we presented a novel algorithm, mopo16S, for optimal primer design in 16S metagenomics experiments. Primers are optimized according to three criteria, namely efficiency of the primer sets, coverage of the representative set and coverage bias across the representative set.

Both the representative set of sequences to be covered and the initial set of state-of-the-art primers are drawn from publicly available and up-to-date databases. Thus, new solutions can always be aligned with the current knowledge on the 16S gene.

In our study, we selected primers that could generate relatively long amplicons because we wanted to include several variable regions of the 16S rRNA gene, in order to improve the ability to taxonomically classify bacterial sequences (OTU) at genera or even species level [32]. Please note, however, that mopo16S is general enough to be applicable to any desired amplicon length. Of note, amplicon length is not affecting the computational performance of the algorithm, as the search for the optimal solution is performed in the space of primers. Only the parameters related to the amount of effort in searching for the optimal solution (i.e. the number of runs of the MULTI-OBJECTIVE-SEARCH algorithm and the number of restarts *n*_*rest*_ of each run) can affect the execution time of the software. To mitigate this effect, mopo16S executes each run of the MULTI-OBJECTIVE-SEARCH algorithm on a different thread, resulting in an execution time speed-up that is almost linear in the number of threads used.

To solve the multi-objective optimization problem we chose to use a local search approach rather than a population-based search algorithm, such as a multi-objective evolutionary algorithm, for several reasons. First, the nature of our search space, i.e. the space of all possible primer pairs, lends itself naturally to a local search paradigm, where the effect of changing, adding or dropping one base at a time starting from an initially good solution (the literature primer pairs) is often not harnessing much the primer feasibility. On the other hand, we reckon that the recombination operator typical of genetic algorithms used to combine two parent solutions [33, 34] would almost often result in unfeasible primers, slowing down the search for the optimum. Second, the strength of local search is the scarcity of parameters to be tuned. In particular, for single-objective local search we chose the *iterated best improvement local search* approach, which is parameter-less and terminates when no further improvement is found. On the opposite, evolutionary algorithms, compared to local search, have many more parameters that need to be accurately tuned and that, even when optimally tuned for a set of instances, do not guarantee to remain optimal for unseen data.

## Conclusions

Many of the current bacterial 16S primers have been designed from sequence data obtained from in vitro cultured species, even though only a minority of bacterial species can be cultured in the laboratory. However, our knowledge of unculturable bacteria sequences is rapidly growing thanks to NGS and several 16S sequence databases have been created and are being maintained up to date by the scientific community. There is thus the need for automated methods to design and update bacterial 16S primers able to take into account such new available information.

In this work, we give our contribution to the field by presenting a method for optimal multi-objective primer choice, which exploits publicly available databases such as GreenGenes [3], probeBase [15, 16] and SILVA [5]. mopo16S can be applied to any desired amplicon length and representative bacteria population. Our approach:

- Maximizes experimental efficiency and specificity, in terms of how much a primer pair is able to amplify the selected DNA sequence during PCR.
- Maximizes coverage, in terms of the fraction of all bacterial 16S sequences from different species that are matched by at least one forward and one reverse primer from the set pair.
- Minimizes matching-bias, in terms of differences in the number of combinations of primers from the forward and reverse sets matching each bacterial 16S.

We developed a software tool implementing our approach and released it under the GNU General Public Licence as the mopo16S software tool (Multi-Objective Primer Optimization for 16S experiments) at http://sysbiobig.dei.unipd.it/?q=Software#mopo16S.

We tested mopo16S on an example problem: the optimal primers choice for Bacterial 16S and amplicons in the range of 700–800 bp. The three resulting primer-set-pairs, when assessed in silico, outperformed state-of-the-art primers according to all three optimization criteria. Experimentally, the three PCR primer sets were demonstrated to be suitable for the amplification of 16S rRNAs from a variety of bacterial species belonging to different genera, thus confirming the predicted efficiency, wide coverage and low matching-bias.

## Additional file

Additional file 1: **Table S1.** Supplementary information on the identification of bacterial 16S sequences and experimental performance. (DOCX 610 kb)

## Abbreviations

NGS: Next Generation Sequencing
OTUs: Operational Taxonomic Units
PCR: Polymerase Chain Reaction
rRNA: Ribosomal RNA

## References

[1] Kuczynski J, Lauber CL, Walters WA, Parfrey LW, Clemente JC, Gevers D, Knight R (2012). Experimental and analytical tools for studying the human microbiome. Nat Rev Genet.

[2] Finotello F, Mastrorilli E, Di Camillo B. Measuring the diversity of the human microbiota with targeted next-generation sequencing. Brief Bioinform. 2016. 10.1093/bib/bbw119. 10.1093/bib/bbw119 28025179

[3] Kircher M, Kelso J (2010). High-throughput DNA sequencing – concepts and limitations. BioEssays.

[4] DeSantis TZ, Hugenholtz P, Larsen N, Rojas M, Brodie EL, Keller K, Huber T, Dalevi D, Hu P, Andersen GL (2006). Greengenes, a chimera-checked 16S rRNA gene database and workbench compatible with ARB. Appl Environ Microbiol.

[5] Cole JR, Wang Q, Fish JA, Chai B, McGarrell DM, Sun Y, Brown CT, Porras-Alfaro A, Kuske CR, Tiedje JM (2014). Ribosomal database project: data and tools for high throughput rRNA analysis. Nucleic Acids Res.

[6] Quast C, Pruesse E, Yilmaz P, Gerken J, Schweer T, Yarza P, Peplies J, Glöckner FO (2013). The SILVA ribosomal RNA gene database project: improved data processing and web-based tools. Nucleic Acids Res.

[7] Dieffenbach CW, Lowe TM, Dveksler GS (1993). General concepts for PCR primer design. Genome Res.

[8] Linhart C, Shamir R (2002). The degenerate primer design problem. Bioinformatics.

[9] Hugerth LW, Wefer HA, Lundin S, Jakobsson HE, Lindberg M, Rodin S, Engstrand L, Andersson AF (2014). Degeprime, a program for degenerate primer design for broad-taxonomic-range pcr in microbial ecology studies. Appl Environ Microbiol.

[10] Brodin J, Krishnamoorthy M, Athreya G, Fischer W, Hraber P, Gleasner C, Green L, Korber B, Leitner T (2013). A multiple-alignment based primer design algorithm for genetically highly variable dna targets. BMC Bioinformatics.

[11] Feng D, Doolittle R (1987). Progressive sequence alignment as a prerequisite to correct phylogenetic trees. J Mol Evol.

[12] Wang Y, Qian PY (2009). Conservative fragments in bacterial 16S rRNA genes and primer design for 16S ribosomal DNA amplicons in metagenomic studies. PLoS One.

[13] Thomas MC, Thomas DK, Selinger LB, Inglis GD (2011). SPYDER, a new method for in silico design and assessment of 16S rRNA gene primers for molecular microbial ecology. FEMS Microbiol Lett.

[14] Gravitt PE, Peyton CL, Alessi TQ, Wheeler CM, Coutlee F, Hildesheim A, Schiffman MH, Scott DR, Apple RJ (2000). Improved amplification of genital human papillomaviruses. J Clin Microbiol.

[15] Loy A, Maixner F, Wagner M, Horn M (2007). probeBase - an online resource for rRNA-targeted oligonucleotide probes: new features 2007. Nucleic Acids Res.

[16] Klindworth A, Pruesse E, Schweer T, Peplies J, Quast C, Horn M, Glöckner FO (2013). Evaluation of general 16S ribosomal RNA gene PCR primers for classical and next-generation sequencing-based diversity studies. Nucleic Acids Res.

[17] SantaLucia J, Allawi HT, Seneviratne PA (1996). Improved nearest-neighbor parameters for predicting DNA duplex stability. Biochemistry.

[18] Apte A., Daniel S. (2009). PCR Primer Design. Cold Spring Harbor Protocols.

[19] JM R, Walsh-Weller J (1998). An introduction to PCR primer design and optimization of amplification reactions. Methods Mol Biol.

[20] Lefever S, Pattyn F, Hellemans J, Vandesompele J (2013). Single-nucleotide polymorphisms and other mismatches reduce performance of quantitative PCR assays. Clin Chem.

[21] Acland A, Agarwala R, Barrett T, Beck J, Benson DA, Bollin C, Bolton E, Bryant SH, Canese K, Church DM, Clark K (2014). Database resources of the national center for biotechnology information. Nucleic Acids Res.

[22] Paquete Luis, Stutzle Thomas (2007). Stochastic Local Search Algorithms for Multiobjective Combinatorial Optimization. Handbook of Approximation Algorithms and Metaheuristics.

[23] Dubois-Lacoste J, López-Ibáñez M, Stützle T (2011). Improving the anytime behavior of two-phase local search. Ann Math Artif Intell.

[24] Sambo F, Borrotti M, Mylona K (2014). A coordinate-exchange two-phase local search algorithm for the D- and I-optimal designs of split-plot experiments. Comput Stat Data Anal.

[25] Borrotti M, Sambo F, Mylona K, Gilmour S (2017). A multi-objective coordinate-exchange two-phase local search algorithm for multi-stratum experiments. Stat Comput.

[26] Hoos HH, Stǘtzle T. Stochastic Local Search: Foundations & Applications. San Francisco: Elsevier; 2004.

[27] Döring A, Weese D, Rausch T, Reinert K (2008). SeqAn an efficient, generic C++ library for sequence analysis. BMC Bioinformatics.

[28] The OpenMP API specification for parallel programming. 2013. https://www.openmp.org/. Accessed 1 Mar 2017.

[29] Pearson WR, Lipman DJ (1988). Improved tools for biological sequence comparison. Proc Natl Acad Sci U S A.

[30] Shimodaira H (1998). An application of multiple comparison techniques to model selection. Ann Inst Stat Math.

[31] Shimodaira H, Hasegawa M (1999). Multiple comparisons of log-likelihoods with applications to phylogenetic inference. Mol Biol Evol.

[32] Franzén O, Hu J, Bao X, Itzkowitz SH, Peter I, Bashir A (2015). Improved OTU-picking using long-read 16S rRNA gene amplicon sequencing and generic hierarchical clustering. Microbiome.

[33] Zhang Q, Li H (2007). MOEA/D: a multi-objective evolutionary algorithm based on decomposition. IEEE Trans Evol Comput.

[34] Knowles JD, Corne DW (2000). Approximating the nondominated front using the Pareto archived evolution strategy. Evol Comput.

