# Supplementary material for: Optimizing PCR primers targeting the bacterial 16S ribosomal RNA gene
Source: BMC Bioinformatics. 2018 Sep 29;19:343. doi: 10.1186/s12859-018-2360-6 (PMC6162885; doi:10.1186/s12859-018-2360-6)
Supplement: Supplementary file 1 — Table S1. Supplementary information on the identification of bacterial 16S sequences and experimental performance. (DOCX 610 kb) [file 12859_2018_2360_MOESM1_ESM.docx]

# Robust identification of bacterial 16S sequences

To robustly identify only bacterial 16S in the GreenGenes annotation, discarding sequences possibly belonging to archaea, eukaryotes or viruses, we leveraged on the NCBI taxonomy [1].

However, since domain information is missing from NCBI annotation for around 20% of the sequences (called *unknown* sequences from here on), we designed an *ad hoc* procedure to re-annotate each of these sequences. We compared all 16S sequences with the *Escherichia coli* 16S (Genbank: AX039566.1), here used as model organism, using the *ssearch36* function of the fasta-36 package [2]. We scored each alignment considering the following indices:

- **Aid:** percentage identity; see BLAST Glossary [3];
- **Mism:** number of mismatches;
- **Gap:** number of gap nucleotides;
- **E value:** transformed to log-scale; see BLAST Glossary [3];
- **Bit score:** see BLAST Glossary [3];
- **Alen1:** ratio between alignment length and 16S sequence length;
- **Alen2:** ratio between the portion of 16S sequence aligned and total 16S length.

Secondary alignments with Aid<50% were discarded, in order to keep only the best alignment for each sequence. The annotated sequences were used to identify the values of the seven indices defined above that separate Bacteria from Archaea, minimizing false positives (FPs), i.e. Archaea classified as Bacteria, and false negative (FNs), i.e. Bacteria classified as Archaea. In particular, for each index, we identified a condition and a threshold that guarantee FPs=FNs (Table S1).

**Table S1.** Index conditions and thresholds for the identification of bacterial 16S.

| **Index** | **Condition** | **Threshold** |
| --- | --- | --- |
| Aid | greater | 64.51 |
| Mism | less | 410 |
| Gap | less | 87.35 |
| E value | less | -140.4 |
| Bit score | greater | 479 |
| Alen1 | greater | 0.99 |
| Alen2 | greater | 0.95 |

The identified conditions were then used to classify the unknown sequences: 92% of the unknown sequences satisfied the criteria of Supplementary Table S1 and were assigned to the Bacteria domain.

Finally, to select bacteria representative sequences from the database, we conservatively considered only the sequences annotated as such both in our curated NCBI-based annotation and in the original GreenGenes annotation. This resulted in a set of 4573 representative 16S sequences belonging to the Bacteria domain from the GreenGenes 85% representative set.

# Reference

[1] Acland A, Agarwala R, Barrett T, Beck J, Benson DA, Bollin C, Bolton E, Bryant SH, Canese K, Church DM, Clark K. Database resources of the National Center for Biotechnology Information. *Nucleic Acids Research*. 2014;42(D1):D7--D17

[2] Pearson WR, Lipman DJ. Improved tools for biological sequence comparison. Proc. Natl. Acad. Sci. U.S.A. 1988;85(8):2444–2448.

[3] Fassler J, Cooper P. Blast glossary. 2011. <http://www.ncbi.nlm.nih.gov/books/NBK62051/> Accessed 1 Sep 2016.

# Experimental performance

Performance of the three primer sets was evaluated in a panel of bacteria isolated from clinical specimens, including representatives of common gram-positive and gram-negative human pathogens belonging to different genera and phyla (Table S2).

**Supplementary Table S2.** Bacteria species amplified by real-time PCR using the three 16S rRNA primer sets identified by mopo16S and three non-optimized primer sets (Forward: Bact-0008-b-S-2 - Reverse: Bact-0785-a-A-21; Forward: Bact-0347-a-S-19; Reverse: Bact-1028-b-A-19; Forward: Bact-0337-a-S-20; Reverse: Bact-1046-a-A-19).

|  | **Phylum** | **Family** | **Genus** | **Species** |
| --- | --- | --- | --- | --- |
| 1 | Actinobacteria | *Bifidobacteriaceae* | *Gardnerella* | *Gardnerella vaginalis* |
| 2 | Actinobacteria | *Micrococcaceae* | *Micrococcus* | *Micrococcus luteus* |
| 3 | Actinobacteria | *Micrococcaceae* | *Rothia* | *Rothia mucilaginosa* |
| 4 | Bacteroidetes | *Bacteroidaceae* | *Bacteroides* | *Bacteroides stercoris* |
| 5 | Bacteroidetes | *Bacteroidaceae* | *Bacteroides* | *Bacteroides fragilis* |
| 6 | Bacteroidetes | *Bacteroidaceae* | *Bacteroides* | *Bacteroides fragilis* |
| 7 | Bacteroidetes | *Prevotellaceae* | *Prevotella* | *Prevotella timonensis* |
| 8 | Fusobacteria | *Fusobacteriaceae* | *Fusobacterium* | *Fusobacterium nucleatum* |
| 9 | Fusobacteria | *Fusobacteriaceae* | *Fusobacterium* | *Fusobacterium necroforum* |
| 10 | Firmicutes | *Peptostreptococcaceae* | *Clostridioides* | *Clostridioides difficile* |
| 11 | Firmicutes | *Bacillaceae* | *Bacillus* | *Bacillus licheniformis* |
| 12 | Firmicutes | *Lactobacillaceae* | *Lactobacillus* | *Lactobacillus salivarius* |
| 13 | Firmicutes | *Staphylococcaceae* | *Staphylococcus* | *Staphylococcus capitis* |
| 14 | Firmicutes | *Staphylococcaceae* | *Staphylococcus* | *Staphylococcus aureus* |
| 15 | Firmicutes | *Staphylococcaceae* | *Staphylococcus* | *Staphylococcus caprae* |
| 16 | Firmicutes | *Staphylococcaceae* | *Staphylococcus* | *Staphylococcus saprophyticus* |
| 17 | Firmicutes | *Staphylococcaceae* | *Staphylococcus* | *Staphylococcus epidermidis* |
| 18 | Firmicutes | *Enterococcaceae* | *Enterococcus* | *Enterococcus faecium* |
| 19 | Firmicutes | *Enterococcaceae* | *Enterococcus* | *Enterococcus avium* |
| 20 | Firmicutes | *Streptococcaceae* | *Streptococcus* | *Streptococcus constellatus* |
| 21 | Firmicutes | *Streptococcaceae* | *Streptococcus* | *Streptococcus dysgalactiae* |
| 22 | Firmicutes | *Streptococcaceae* | *Streptococcus* | *Streptococcus anginosus* |
| 23 | Firmicutes | *Streptococcaceae* | *Streptococcus* | *Streptococcus intermedius* |
| 24 | Firmicutes | *Streptococcaceae* | *Streptococcus* | *Streptococcus agalactiae* |
| 25 | Proteobacteria | *Enterobacteriaceae* | *Serratia* | *Serratia marcescens* |
| 26 | Proteobacteria | *Enterobacteriaceae* | *Klebsiella* | *Klebsiella pneumoniae* |
| 27 | Proteobacteria | *Enterobacteriaceae* | *Escherichia* | *Escherichia coli* |
| 28 | Proteobacteria | *Enterobacteriaceae* | *Proteus* | *Proteus mirabilis* |
| 29 | Proteobacteria | *Enterobacteriaceae* | *Raoultella* | *Raoultella planticola* |
| 30 | Proteobacteria | *Enterobacteriaceae* | *Escherichia* | *Escherichia coli* |
| 31 | Proteobacteria | *Moraxellaceae* | *Acinetobacter* | *Acinetobacter baumannii* |
| 32 | Proteobacteria | *Moraxellaceae* | *Acinetobacter* | *Acinetobacter lwoffii* |
| 33 | Proteobacteria | *Pasteurellaceae* | *Haemophilus* | *Haemophilus influenzae* |
| 34 | Proteobacteria | *Pseudomonadaceae* | *Pseudomonas* | *Pseudomonas aeruginosa* |
| 35 | Proteobacteria | *Xanthomonadaceae* | *Stenotrophomonas* | *Stenotrophomonas maltophilia* |
| 36 | Proteobacteria | *Helicobacteraceae* | *Helicobacter* | *Helicobacter pylori* |

Figure S1: Amplification of 16S rRNA by real-time PCR based on SYBR-Green I fluorescence. Amplification plots (upper panels) and dissociation curves (lower panels) of three representative bacteria samples and three negative controls. Human genomic DNA was included in the negative controls.
